# Using Twitter to Understand COVID-19 Vaccine-Related Ageism During the Pandemic

**DOI:** 10.1093/geront/gnad061

**Published:** 2023-06-02

**Authors:** Juanita-Dawne R Bacsu, Melissa K Andrew, Mehrnoosh Azizi, Corinne Berger, Allison Cammer, Alison L Chasteen, Sarah Anne Fraser, Karl S Grewal, Shoshana Green, Rory Gowda-Sookochoff, Jasmine Cassy Mah, Katherine S McGilton, Laura Middleton, Kate Nanson, Raymond J Spiteri, Yikai Tang, Megan E O’Connell

**Affiliations:** School of Nursing, Thompson Rivers University, Kamloops, British Columbia, Canada; Division of Geriatric Medicine, Dalhousie University, Halifax, Nova Scotia, Canada; Department of Computer Science, University of Saskatchewan, Saskatoon, Saskatchewan, Canada; Department of Computer Science, University of Saskatchewan, Saskatoon, Saskatchewan, Canada; College of Pharmacy and Nutrition, University of Saskatchewan, Saskatoon, Saskatchewan, Canada; Department of Psychology, University of Toronto, Toronto, Ontario, Canada; Interdisciplinary School of Health Sciences, Faculty of Health Sciences, University of Ottawa, Ottawa, Ontario, Canada; Department of Psychology, University of Saskatchewan, Saskatoon, Saskatchewan, Canada; Department of Psychology, University of Saskatchewan, Saskatoon, Saskatchewan, Canada; Department of Psychology, University of Saskatchewan, Saskatoon, Saskatchewan, Canada; Department of Medicine, Dalhousie University, Halifax, Nova Scotia, Canada; KITE Research Institute, Toronto Rehabilitation Institute, University Health Network, Toronto, Ontario, Canada; Kinesiology and Health Studies, University of Waterloo, Waterloo, Ontario, Canada; School of Nursing, Thompson Rivers University, Kamloops, British Columbia, Canada; Department of Computer Science, University of Saskatchewan, Saskatoon, Saskatchewan, Canada; Department of Psychology, University of Toronto, Toronto, Ontario, Canada; Department of Psychology, Canadian Centre for Health and Safety in Agriculture, University of Saskatchewan, Saskatoon, Saskatchewan, Canada

**Keywords:** Attitudes, Discrimination, SARS-CoV-2, Social media, Stereotypes

## Abstract

**Background and Objectives:**

During the rollout of coronavirus 2019 (COVID-19) vaccines, older adults in high-income countries were often prioritized for inoculation in efforts to reduce COVID-19-related mortality. However, this prioritization may have contributed to intergenerational tensions and ageism, particularly with the limited supply of COVID-19 vaccines. This study examines Twitter discourse to understand vaccine-related ageism during the COVID-19 pandemic to inform future vaccination policies and practices to reduce ageism.

**Research Design and Methods:**

We collected 1,369 relevant tweets on Twitter using the Twint application in Python from December 8, 2020, to December 31, 2021. Tweets were analyzed using thematic analysis, and steps were taken to ensure rigor.

**Results:**

Our research identified four main themes including (a) blame and hostility: “It’s all their fault”; (b) incompetence and misinformation: “clueless boomer”; (c) ageist political slander; and (d) combatting ageism: advocacy and accessibility.

**Discussion and Implications:**

Our findings exposed issues of victim-blaming, hate speech, pejorative content, and ageist political slander that is deepening the divide of intergenerational conflict. Although a subset of tweets countered negative outcomes and demonstrated intergenerational solidarity, our findings suggest that ageism may have contributed to COVID-19 vaccine hesitancy among older adults. Consequently, urgent action is needed to counter vaccine misinformation, prohibit aggressive messaging, and promote intergenerational unity during the COVID-19 pandemic and beyond.

## Background and Objectives

The coronavirus 2019 (COVID-19) pandemic has exacerbated issues of ageism toward older adults. Existing studies have found that ageism (such as negative stereotypes, beliefs, and discrimination toward older adults) has become rampant on social media platforms (Graham, 2022; Meisner, 2021; Ng & Indran, 2022), including Twitter. A recent report by the World Health Organization and the United Nations (2021) notes that ageism leads to reduced mental and physical health, social isolation, early mortality, and costs society billions of dollars every year. Moreover, Rowe (2022) describes that a new era of COVID-19 ageism has emerged from false rhetoric claiming that pandemic prevention and medical treatment of older adults are futile and a lost cause.

Several studies using Twitter data have examined ageism toward older adults during the COVID-19 pandemic (Barrett et al., 2021; Jimenez-Sotomayor et al., 2020; Ng et al., 2022; Sipocz et al., 2021; Skipper & Rose, 2021; Spaccatini et al., 2022). These studies have provided insight into social media discourse regarding age-based discrimination (Spaccatini et al., 2022), stereotypes (homogenizing older adults as frail, vulnerable, and requiring protection; Ng et al., 2022), political ageism (Skipper & Rose, 2021), jokes, ridicule, false information, devaluing the lives of older adults (Bacsu et al., 2022a; Jimenez-Sotomayor et al., 2020), and intergenerational unity (Barrett et al., 2021; Sipocz et al., 2021).

Although these studies shed light on COVID-19 ageism, most of this research only examines the early stages of the pandemic (Barrett et al., 2021; Jimenez-Sotomayor et al., 2020; Sipocz et al., 2021; Skipper & Rose, 2021; Spaccatini, et al., 2022). For example, Barrett et al. (2021) examined tweets posted on 1 day during March 23, 2020, whereas Jimenez-Sotomayor et al. (2020) analyzed tweets over 10 days in March 2020. Consequently, there is a paucity of research analyzing ageism beyond the initial stages of the pandemic, particularly in relation to the major milestone of the COVID-19 vaccination rollout.

During the vaccination rollout, many high-income countries prioritized older adults to receive inoculation in efforts to reduce COVID-19-related mortality (Pullen et al., 2021; World Health Organization, 2023). Emerging research suggests that the limited supply of COVID-19 vaccines and the prioritization of older adults may have contributed to intergenerational tension and ageism (Lloyd-Sherlock et al., 2021; Pullen et al., 2021). Consequently, North and Fiske (2013) note that ageism and intergenerational rivalry are often based on prescriptive age stereotypes that older adults should not consume or overutilize scarce resources such as health care.

Understanding ageism and age-related vaccine sentiments is important to supporting COVID-19 vaccination campaigns and uptake. More specifically, examining vaccine-related ageism on Twitter may help policy-makers and health practitioners address vaccination concerns, fears, and misconceptions to better support vaccination uptake. With over 368 million monthly users (Statista, 2022), Twitter has become a widely used platform to share concerns and beliefs regarding COVID-19 vaccines and ageism. Accordingly, this study examines Twitter discourse to understand COVID-19 vaccine-related ageism during the pandemic to inform future vaccination policies and practices to reduce ageism.

## Research Design and Methods

### Ethics

Our research did not seek ethics approval because our study was based on publicly available information (Takats et al., 2022). More specifically, our research analyzed large volumes of text (i.e., tweets) that were publicly shared on Twitter and are typically not deemed human subject research (JMIR, 2022). To help support the privacy of Twitter users, we omitted the usernames and Twitter handles from our findings. However, we cannot guarantee that our tweets cannot be tracked online to the users. Consequently, emerging research notes that it is important for social media researchers to consider ethical practice beyond procedural ethics (Ravn et al., 2020).

### Data Collection and Sample

Tweets were collected using the advanced scraping tool Twint, written in the Python programming language. Twint can scrape tweets without using Twitter’s application programming interface, and hence it avoids various restrictions imposed by Twitter, such as a limit on the number and frequency of tweets scraped and the requirement of Twitter account (Ambi, 2021). Twint was used to scrape tweets for the period of December 8, 2020, to December 31, 2021. These dates were selected since December 8, 2020, was the first COVID-19 vaccination in the world, with increasing rollout until the end date of the scraping, which was December 2021.

Our search terms included a range of words focused on three concepts: ageist terminology/labels (e.g., boomer, coot, etc.), vaccines, and the COVID-19 pandemic. Our ageist search terms were developed by drawing on an ageist-language glossary published in the American Association of Retired Persons (Duarte & Albo, 2018) and a list of negative terms often used to describe older adults on social media (Centre for Ageing Better, 2021). The full set of our search terms consisted of (“old hag” OR “old codger” OR “sad old” OR “bitter old” OR “old coot” OR “wee granny” OR “grumpy old” OR “boomer remover” OR “most selfish generation ever” OR “old bastard” OR “ancient” OR “oldies” OR “old timer” OR “old senile” OR “senile old” OR “boomer” OR “old fart” OR “codger” OR “coot” OR “geezer” OR “old grumpy” OR “grumpy old” OR “crazy old” OR “old crazy” OR “old sad” OR “old bitter” OR “old biddy” OR “old bag” OR “old bat” OR “old fogey” OR “old grannies”) AND (“Vaccine” OR “vaccinate” OR “vaccination” OR “shot” OR “immunize” OR “needle” OR “jab” OR “stick it” OR “poke”) AND (“corona” OR “covid”).

**Figure 1.**
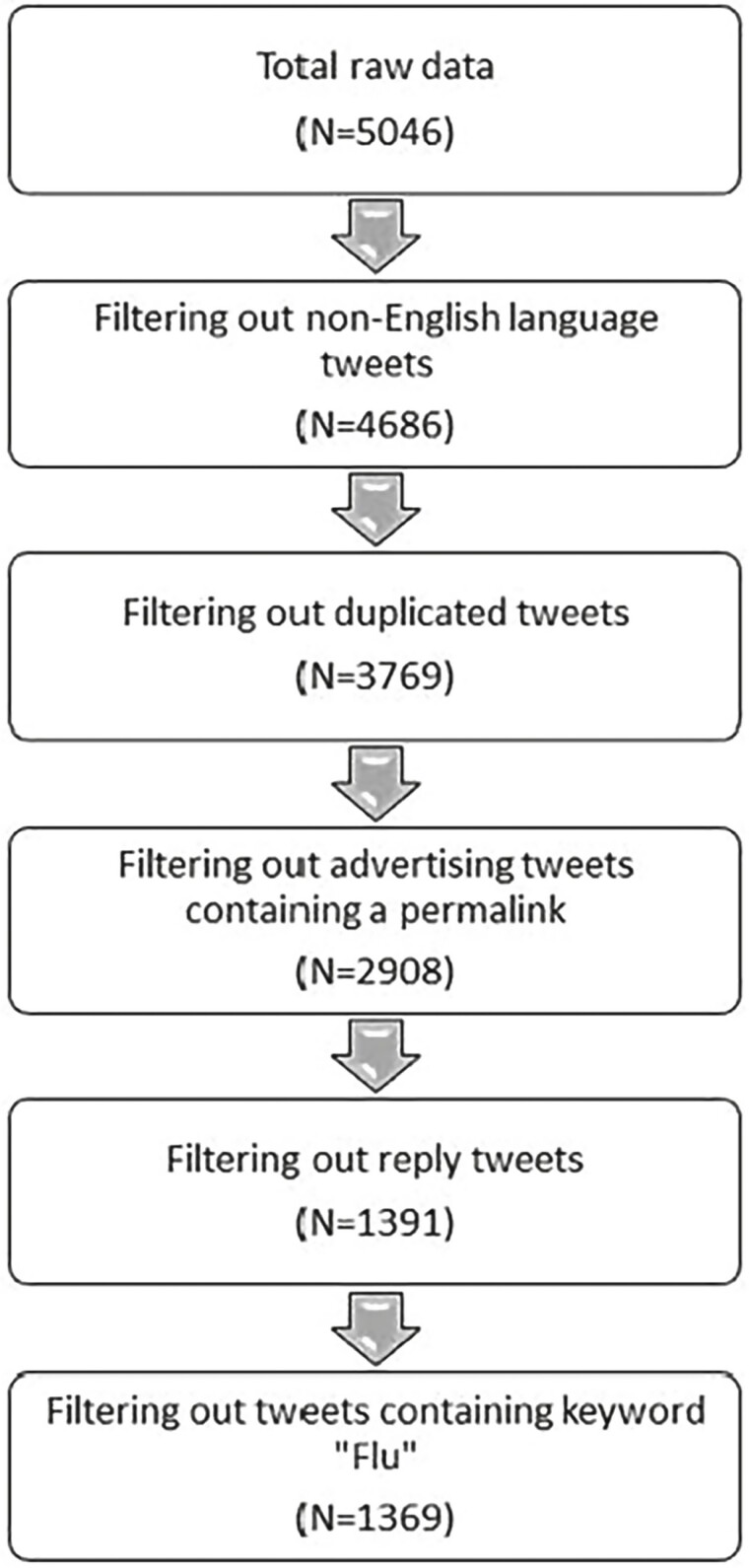
Twitter filtering process.

The initial worldwide search returned 5,046 tweets. Tweets not in English were excluded, along with duplicated tweets, advertisements and spam, replies to tweets, and tweets containing keyword “Flu” (see Figure 1). The team decided to exclude the word “flu” because such tweets often referred to the flu vaccine rather than the COVID-19 vaccine. Replies to tweets were removed because they often were missing information or a large part of the conversation; therefore, only original tweets were cited. The remaining 1,369 tweets were exported for thematic analysis.

### Data Analysis

Tweets were analyzed using an adapted approach that was guided by Braun and Clarke’s (2021) five-stage thematic analysis process including (1) familiarization with the data; (2) generating initial codes by reviewing the data; (3) searching for initial overarching themes; (4) reviewing the themes; and (5) naming the themes. A combination of inductive (data-driven, bottom-up) and deductive (top-down) components was used to analyze the data.

First, an initial coding team was formed with four of the coauthors (J.-D. R. Bacsu, A. Cammer, M. Aziz, R. Gowda-Sookochoff) who had diverse interdisciplinary expertise and backgrounds including community health, nutrition, computer science, and psychology. This team was formed to develop and pilot test a codebook that was created by reviewing tweets (i.e., familiarization) to develop an initial list of the codes. To begin the coding process, an inductive bottom-up (data-driven) approach was used where the researchers started with no codes and created the codes as they analyzed the data. For the pilot test, the coding team independently coded 150 tweets and then compared their codes with each other. A final codebook was created that consisted of seven codes including perpetuating ageism, blame, false information, political ageism, challenging ageism, self-ageism, and “well-intentioned” labeling.

The full team of coauthors participated in two different practice coding activities using a deductive approach (top-down) that was guided by the finalized list of codes (codebook). The first activity involved independently coding 75 tweets and then comparing individual codes to an answer sheet developed by the initial coding team (J.-D. R. Bacsu, A. Cammer, R. Gowda-Sookochoff, M. Aziz). The second practice activity involved a team coding exercise in which the full group collectively coded and discussed any coding uncertainties, challenges, questions, or disagreements for 30 tweets. After the practice coding was completed, eight pairs of coauthors (i.e., 16 coders) independently coded the same tweets (approximately 171 tweets per coder) and then met to compare codes and determine the final code for each tweet through discussion and pair consensus. Any coding discrepancies or disagreements were resolved through group consensus.

Following coding, an inductive approach was used where the research team searched through the data to identify the common themes by placing the codes into overarching themes (Table 1—thematic map). After this step, the full team reviewed the themes to ensure that no themes were missed, the themes were clear and understandable, and the themes were distinguishable from each other (Braun & Clarke, 2006). Next, the team reviewed the theme names through group discussion to confirm the clarity and conciseness of each theme name.

**Table 1.** Search Terms

Concept	Keywords
Ageism	“old hag” OR “old codger” OR “sad old” OR “bitter old” OR “old coot” OR “wee granny” OR “grumpy old” OR “boomer remover” OR”most selfish generation ever” OR “old bastard” OR “ancient” OR”oldies” OR “old timer” OR “old senile” OR “senile old” OR “boomer” OR “old fart” OR “codger” OR “coot” OR “geezer” OR “old grumpy” OR “grumpy old” OR “crazy old” OR “old crazy” OR “old sad” OR “old bitter” OR “old biddy” OR “old bag” OR “old bat” OR “old fogey” OR “old grannies”
Vaccine	“vaccine” OR “vaccinate” OR “vaccination” OR “shot” OR “immunize” OR “needle” OR “jab” OR “stick it” OR “poke”
Coronavirus disease 2019	“corona” OR “Covid”

### Trustworthiness and Rigor

**Figure 2.**
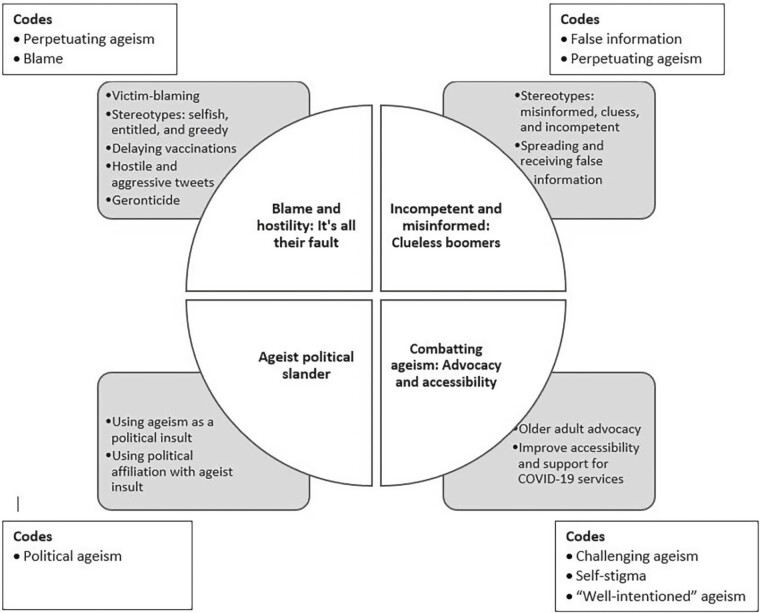
Thematic map.

We used four main strategies to ensure trustworthiness and rigor in our data collection and analysis (O’Conner & Joffe, 2020; Raskind et al., 2019). First, a robust audit trail was kept to ensure trustworthiness by documenting our research methods and tools such as our list of search terms (Table 1), data extraction process (Figure 1), and thematic map (Figure 2). Second, each coder used memoing to make note of any similarities, differences, and initial themes during the coding process (Raskind et al., 2019). Third, we used multiple coding by having each tweet coded independently by two coders to provide cross-checks in the interpretation of the tweets (O’Conner & Joffe, 2020). After this step, we analyzed the intercoder reliability by calculating the percentage of agreement between each of the eight different pairs of coders (i.e., two independent coders for each tweet) and then taking each of the pairs’ percentage of agreement numbers to calculate the overall group average, which was 86% agreement across coded tweet (Table 2). Fourth, our research team consisted of interdisciplinary researchers (i.e., nursing, nutrition, psychology, community health and epidemiology, geriatrics, vaccinology, computer science, and gerontology) with diverse skills and expertise that supported reflexivity and team learning for a more nuanced approach to interpret our study’s findings. More specifically, reflexivity, active reflection, team learning, and robust discussions challenged our thinking about the terminology, tone, and interpretation of each tweet. The full team met regularly to discuss different aspects of the data analysis including coding, cultural nuances and terminology, initial theme development, reviewing the themes, and defining and naming the themes (Raskind et al., 2019). For example, the team discussed the role of cultural labels and stereotypes in reflecting on ageist terminology on Twitter (Gendron et al., 2016). More specifically, ageism (such as ageist terminology, labels, stereotypes, and discriminatory behaviors) may be greatly influenced at the societal level by culture in different countries (Levy, 2009). A recent study found that among 20 countries, the United Kingdom had the highest ageism score, whereas Sri Lanka had the lowest ageism score (Ng & Lim-Soh, 2021). Consequently, the team discussed the need to be mindful of different terminology and language-based discrimination (e.g., granny, elderly, etc.) that may be used to perpetuate ageism on Twitter (Duarte & Albo, 2018; Gendron et al., 2016; Levy, 2009).

**Table 2.** Intercoder Reliability

Coding partners^a^ for thematic analysis	Percentage of agreement
SG and ALC	93%
KSG and MA	86%
AC and JCM	88%
J-DRB and MA	83%
LM and RG-S	93%
CB and KSM	63%
MEO and RJS	98%
SAF and YT	80%
Intercoder reliability average	85.5%

*Notes*: AC = Allison Cammer; ALC = Alison L. Chasteen; CB = Corinne Berger; JCM = Jasmine Cassy Mah; J-DRB = Juanita-Dawne R. Bacsu; KSM = Katherine S. McGilton; KSG = Karl S. Grewal; LM = Laura Middleton; MA = Mehrnoosh Azizi; MEO = Megan E. O’Connell; RG-S = Rory Gowda-Sookochoff; RJS = Raymond J. Spiteri; SAF = Sarah Anne Fraser; SG = Shoshana Green; YT = Yikai Tang.

^a^Each person is represented by their initials.

## Results

Using thematic analysis, four main themes emerged, including (1) blame and hostility: “It’s all their fault”; (2) incompetence and misinformation: “clueless boomers”; (3) ageist political slander; and (4) combatting ageism: advocacy and accessibility.

### Blame and Hostility: “It’s All Their Fault”

Blame was at the core of many ageist tweets during the COVID-19 pandemic. Several tweets depicted blame and hostility toward older adults for taking away vaccination resources and delaying the vaccination process. Many tweets blamed older adults for vaccine shopping and delaying the vaccination process to receive their vaccination brand of choice. Tweets also described hostility and anger toward older adults for receiving the COVID-19 vaccine before younger generations and persons who were working away from home. This hostility may be attributed to prescriptive age stereotypes (perceived expectations for how older adults should act and behave) and the notion that older adults should not overconsume or deplete the limited supply of COVID-19 vaccinations (North & Fiske, 2013). Consequently, intergenerational competition for scarce resources contributed to expressions of hostile ageism toward older adults by younger adults. This sense of blame and hostility is illustrated in the following tweets:

The worst part about being an American is they prioritized old people getting COVID shots and not young people or professionals. Boomers gunna support each other, they got us beat on how they always stick together, even if they’re evil.

Ok the main storylines of Covid twitter today: AZ vaccine appts empty and boomers vaccine shopping…

I’m A Fucking Covid Tester I Shouldn’t Have To Beg For Vaccine Scraps Because Goddamn Boomers Didn’t Show Up To Their Appointments.

Many tweets also blamed older adults for the COVID-19 lockdowns and destroying jobs, stopping social gatherings, and ruining the economy during the pandemic. This reflects a societal shift from benevolent ageism (notion that older adults deserve special policies based on their unique needs associated with their age) to blame-based ageism and intergenerational tension that faults older adults for societal, political, and economic issues (Binstock, 2010; Cary et al., 2017). For example, a Twitter user stated, “I don’t think #boomers should get the stimulus checks, or the COVID vaccine its really all their fault were in the mess.” Moreover, tweets described older adults as being entitled, whiny, and the “most selfish generation ever” during the pandemic. Blame and perceived entitlement are illustrated in the following tweets:

… The young crashed their social lives and the economy so we could hold out for a vaccine. Stop being so entitled. Guaranteed if Covid affected the young and not the old, plenty of boomers wouldn’t want the vaccine or lockdowns.

The covid response has been the most `boomer` thing ever. Think about it. The disease is only a real threat to the old and unhealthy, but the young people have had their jobs destroyed, savings destroyed, and are being forced to get the shots, all of which they never needed …

This boomer at work was complaining that when she was getting her covid shot she had to wait in line ... The epitome of the most selfish generation ever …

Some tweets expressed extreme anger and hate speech toward older adults. Many of these aggressive tweets argued that older adults needed to die in the pandemic.

Wish Covid-19 could be modified such that it will only w*pe off boomers regardless of vaccination.

Wanna listen to the radio, don’t wanna listen the boomers calling in crying about how much they scared of covid. “I’m shielding!” No you just cowering at home till they text you that your vaccine is ready. Some of you greedy entitled fucks need to die.

Dont forget … the outbreaks were caused by entitled Boomers who refused to get the vaccine offered and were waiting for a luxury vaccine choice. Time to let it rip now for people being selfish … #selfishboomers #covid #LetitRip

These aggressive tweets are extremely problematic, conveying violent sentiments and messaging that may normalize abuse and harm against older adults during the COVID-19 pandemic. Consequently, victim-blaming on social media platforms such as Twitter may exacerbate discriminatory sentiments that may increase older adult risk for abuse and harm, especially during the COVID-19 pandemic.

### Incompetence and Misinformation: “Clueless Boomers”

In many tweets, older adults were portrayed as incompetent, misinformed, naïve, or unaware, and in turn were endangering others and themselves due to their belief in falsehoods or conspiracies about the COVID-19 pandemic. Some tweets about vaccines and older adults propagated ageism and focused on misinformation about vaccines, either accusing older adults of spreading false information (i.e., conspiracy theories) or believing false information from the media. The ageist belief that older adults are particularly misinformed, clueless, and are spreading false information was evident in the tweets as older adults were referred to pejoratively as “boomer.” This theme is illustrated in the following tweets:

all boomers seem to do on Twitter is spread covid misinformation or blame young people for the state of things while refusing to get the only vaccine young people cant have thats available in mass quantities …

The magic of ok boomer. If someone is straight up bat shit and tells me I’m an idiot because I believe the covid vaccination works, I can get into a pointless argument or just say ok boomer. No one ever responds to ok boomer. It makes for a more civilized discourse space.

Media and social network channels were perceived as vehicles for older adults receiving and believing false information. Older adults were often stereotyped as being unable to think critically about information presented, and their inability to challenge this information was often situated within the family context. This stereotype of older adults being characterized as incompetent, forgetful, and feeble is highlighted in existing literature (Apriceno et al., 2021; Chasteen, Schwarz, & Park, 2002; Fiske et al., 2002). These ageist sentiments are demonstrated in the following tweets:

Fun fact: your boomer parents are now watching 6+ hours a day of youtube videos claiming that covid vaccines are actually hookworms being injected to “modify your dna with nano-technology

I told my dad yesterday that he shouldn’t believe all the dumbass boomer covid memes he sees on Facebook, and should go to the experts. Then he told me he saw a round table with 8 doctors saying the vaccine was bad. Jfc

Ageism was also prevalent not only speculatively but also specifically among members of the same family because some views came directly from younger members within the family unit. Frustration with a family member was often couched in ageist tropes, and family members were described with ageist terms or dismissively referred to as “boomer.” For example, “Just sitting here listening to my boomer parents spreading conspiracy theories about covid vaccines” and “So my parents are apparently confused by the fact that you need to renew your COVID shots, same as a flu shot. You know, just in case you still think boomers belong anywhere but rotting in a nursing home.” Consequently, these familial sentiments may suggest that ageism expressed by familiar others (such as family members) may be viewed as more acceptable than when it is expressed by strangers (Horhota et al., 2019).

Intergenerational conflict was apparent in many of the tweets. Issues about vaccination and COVID-19 infection seemed to fuel the generational divide. An “us against them” mentality was communicated, where the person tweeting positioned themselves in opposition to the older adult population or employed disparaging generational stereotypes. Examples of tweets that illustrate this are:

Getting the COVID-19 vaccine should require a strong work ethic so boomers would be obsessed with it.

I’m sorry but who is gonna tell the boomers you can still get covid even if you have the vaccine.

In contrast, there were also tweets from older adults directly challenging the ageist tropes and employing generational experiences to challenge vaccine conspiracies.

I’m a geezer, am I gonna get the Covid vaccine? ABSOLUTELY! In the 50s I stood in line in my school cafeteria for the Polio Vaccine on a sugar cube. Didn’t want to be in an iron lung ... now I don’t want to be on a ventilator. #GetVaccinated #WearAMask.

### Ageist Political Slander

Another theme identified was ageist political slander that fueled intergenerational conflict and tensions. Many of these tweets used ageist language to direct insults at political leaders.

The sad thing is a rotten Old Fart like Trump HAD Covid, had a million dollar treatment that no one else is getting, GOT the vaccine BUT is still promoting the poison of antivaxx propaganda for no reason but to seed division.

AMERICANS R STUPID right DUMBAZZED PELOUSI you 80 year old bag woman.

These ageist political tweets often questioned the competency and credibility of the politicians. Specifically, these tweets used ageist phrases such as “senile old man,” “old crazy guy,” and “addled old coot” to attack the intellect of the politicians. Moreover, the use of age-related insults not only aimed to undermine the politician but also their COVID-19 policies.

Think about Biden’s Afghanistan policy when considering his COVID mask/vaccine policies ... This dementia addled old coot has no idea what ramifications his wholly partisan political actions can/have/will cause.

Can you imagine if Biden wasn’t handed 3 vaccines on a silver platter? The senile old man can’t even find the entrance to the WH. How is he supposed to be in charge of protecting the American people from Covid? #DeltaVariant

In rare instances, ageist language was used but not in an insulting manner. In one instance, the same ageist language was used by two tweeters to show support and contempt for Joe Biden, suggesting context matters; “The old geezer joe Biden rocks ... thanks for Mentioning that $nvax is near to release their covid vaccine!!” In comparison to, “So they interrupt programming nationally to show geezer @joeBiden get his shot for Covid …”

Some tweets made ageist remarks targeting certain political parties and partisan affiliations. Specifically, these tweets often used political affiliations as insults. This form of political ageism is captured in the following tweets:

I just wept in front of conservative BOOMERS getting my COVID vaccine.

hey should’ve called the covid vaccine “free antivirus pro” so republican boomers would actually take it.

Some tweets suggested that those in politics are older and tend to be out of touch with the concerns of younger generations. However, these tweets are problematic because they contribute to the intergenerational divide and the notion of “us” versus “them.”

Biden didn’t address the country in prime-time. He recited his lame covid plan during the day to boomers and the older gen, which is a snub to students and working people. It’s no wonder there’s no trust in the vaccines. Our leaders won’t risk the scrutiny of talking to us.

According to senile old Biden covid is not the menace its FACEBOOK that’s doing the killing by NOT censoring misgivings about vaccine they are slaughtering innocent Americans everyday he’s thinking about sending his justice Dept lapdogs to proceed with charges

How do we oust a sociopathic narcissist and inaugurate an ancient democratic centrist (at best) who has done HORRIFIC things leaves us all in limbo. NOTHING has changed. Where is the stimulus for families and small businesses? When will there be COVID shots. ZERO communication.

Collectively, these tweets use ageist political slander to negatively depict politicians and partisan affiliations. With ageism weaponized against politicians and their political parties, it appears to be another tool to polarize discourse related to the COVID-19 pandemic. Moreover, these ageist political tweets further perpetuate stereotypes and false assumptions toward older adults.

### Combatting Ageism: Advocacy and Accessibility

Combatting perceived ageism was an emergent theme across the included tweets. Statements challenging ageist practices or cheerleading older adults who were among the first to be vaccinated against COVID-19 exemplified this theme. Identifying and condemning vaccine-related policies that perpetuated ageism was a common method to combat ageism. Although many jurisdictions used vaccine guidelines that were based on the best science at the time, there was a perception among older adults that a less preferred vaccine was being offered to older adults because they were somehow “less than” or more expendable.

Give all us oldies the cheapest AZ COVID vaccine, offer youngsters a more expensive choice. Oldies have rights.

The Vaccine for Covid-19 the best appears to be the one from Pfizer, that is the one the PM and some of his Ministers, but us oldies at 85, can only have Astravenica, is this fair to us. It is more expensive, we have get the Cheapo. Gov. don’t know where they are going.

Is it just me? Is this ageism that is occurring over the rollout of the COVID vaccine lost on the rest of Australia? Second rate AZ for the oldies (dont care If you die from DVT), top line Pfizer for the rest because they will pay tax longer.

Suboptimal public-facing communication and understanding of the nuanced and evolving recommendations (e.g., as evidence about safety and effectiveness became available) for the use of certain vaccine products according to age and comorbidity profiles may have contributed to these expressions of frustration about vaccine allocation policies. Others highlighted vaccine rollout programs that were inherently ageist in their failure to consider age-based differences in access to the technology needed to book vaccine appointments. Several tweets described instances of intergenerational altruism—in the form of family and friends providing technological support—that helped overcome this accessibility barrier for certain older adults.

The CVS COVID vaccine booking process is awful and not Boomer friendly. Walgreens isn’t perfect but they have a much better system.

I’m hearing today from A LOT of people trying to get their age 75+ parents, grandparents, aunts and uncles in line for a COVID vaccine. It seems that by the state adding 65-74-year-olds, a bunch of tech-savvy Boomers (1M of them) have beaten their elders in a vaccine stampede ...

Millennials now moving on from begging their Boomer parents to take COVID seriously to refreshing poorly designed DPH websites trying to book them vaccine appointments.

Spending my day signing up oldies for the COVID-19 vaccine. My 90 yo neighbor told me she doesn’t have a computer and cant stand in a line. enacting my PTO to take her, btw. Worry not.

Cheerleading as a method to combat ageism was evident from some tweeters who expressed gratitude that older adults were given access to the vaccine earlier than other age groups or with tweets that expressed a general relief that older adults were beginning to get vaccinated.

I would still prefer wearing mask and will wait for sometime for the vaccine. Not that I am against or have trust issue, its just that I have had COVID and have antibody plus my immune system knows COVID. I can wait, oldies deserve it as it can save them from dying.

I just saw one of my mom’s boomer friends post about wanting to get the Covid vaccine. And so many of her friends publicly stated they were going to get it too! So happy!! Good job, olds!! Yay science!!

Thank you to all the baby boomers posting selfies getting your COVID-19 shots. It’s having an impact on the holdouts, or at least one in my life. #PositivePeerPressure

It is noteworthy that although the above categories of tweets advocate for older people, ageist terms like “boomer” and “oldies” are still omnipresent.

## Discussion and Implications

This study examined Twitter discourse on vaccine ageism during the COVID-19 pandemic. In comparison to earlier research that evaluated ageist COVID-19 tweets at the beginning of the pandemic (Jimenez-Sotomayor et al., 2020; Sipocz et al., 2021; Skipper & Rose, 2021; Spaccatini et al., 2022; Xiang et al., 2021), our study focused on analyzing ageism in the context of the COVID-19 vaccination rollout. Our research identified four main themes related to vaccine ageism including (1) blame and hostility: “it’s all their fault”; (2) incompetence and misinformation: “clueless boomer”; (3) ageist political slander; and (4) combatting ageism: advocacy and accessibility.

Existing research suggests that ageist discourse has had a significant influence on shaping policy and practice (Centre for Ageing Better, 2021; Ng et al., 2021). For instance, our study found that ageist stereotypes on Twitter depicted older adults as being selfish, incompetent, and often blamed as being a nuisance to society (e.g., blamed for COVID-19 economy, social isolation, physical distancing, etc.) who need to be “wiped out” (e.g., “let it rip,” geronticide, etc.). Elliot (2022) suggested that a possible justification for this hostility is the “boomer remover” trend and the perceived blame toward older adults for their failure to address global issues such as climate change. However, these violent tweets are extremely problematic because they often depict death as the resolution, leaving little room for meaningful discussion on equitable policy or practical resolutions. Research shows that death of an older adult is often viewed as less tragic and unjust than that of a younger adult (Chasteen & Madey, 2003). Consequently, how we view and discuss death of older adults impacts how policy-makers, health care workers, and the public value and respect the lives of older adults (Bacsu et al., 2022a).

Understanding COVID-19 vaccine-related ageism provides pertinent insight into informing future vaccination campaigns and inoculation policies. Specifically, our study’s findings suggest that ageism may have contributed to fear and COVID-19 vaccine hesitancy among older adults. Older adults described having mistrust toward different brands of vaccines related to fears of structural ageism in the COVID-19 vaccination rollout. For example, there were concerns that older adults were being offered cheaper vaccines with more side effects to save the more effective vaccines for younger adults (e.g., “Second rate AZ for the oldies … Top line Pfizer for the rest … Give all oldies the cheapest AZ Covid vaccine ...”). However, many tweets blamed older adults for vaccine shopping thereby delaying the vaccination process, rather than addressing the more complex issues of structural ageism that may have contributed to older adults’ fear and mistrust toward the COVID-19 vaccinations.

Our findings showed that vaccine ageism on Twitter was often widespread and considered acceptable discourse. More specifically, our study found that Twitter was used to disseminate ageist content such as victim-blaming, hate speech, and pejorative discourse about older adults in the pandemic. The anonymity of social media enables users to assert hostile sentiments and victim-blaming with no accountability or repercussions. However, this social media discourse dehumanizes older adults and can influence public perceptions, beliefs, and actions toward older adults. Moreover, ageist discourse and hate speech on social media platforms such as Twitter normalize age-based verbal aggression that increases older adults’ risk for abuse and harm, especially during the pandemic.

During the pandemic, there have been increasing human rights violations against older adults (Bacsu et al., 2022b; Previtali et al., 2020; Rowe, 2022; Swift & Chasteen, 2021). In many countries, age has been used as the only criterion for access to medical services, lifesaving supports, and for physical isolation (WHO, 2021). In Italy, a report by Amnesty International Italia (2022) documents numerous human rights violations against older adults including health care discrimination and the refusal of hospitals to admit residents of long-term care facilities with flu-like symptoms. Moreover, many governments enacted nation-wide lockdowns that directly affected older adults by significantly reducing access to health care services and supports (e.g., meal programs, safe housing, public transportation, etc.) required to subsist during the pandemic (Fraser et al., 2020). Accordingly, ageist sentiments and beliefs promote structural inequities in COVID-19 policy and practice leading to dire consequences for older adults.

Our findings also highlighted issues of ageism within the family unit. For example, intergenerational conflict and tension (frustration, annoyance, and dismissive remarks) were prevalent among members of the same family. Although it is difficult to ascertain whether this conflict existed prior to COVID-19 vaccination policies, the blatant expression of annoyance toward familial older adults highlights issues of disrespect and ageism within the family unit. As Wilkerson (2021) notes, an ageist caste system exists where older adults are devalued, demeaned, and receive second-class status whereby their lives are often viewed as being less worthy than those of younger generations. It is up to society to challenge these ageist notions, beliefs, and values and focus on what generations have in common. Not perpetuating an ageist caste system and learning from other cultures that respecting older adults may be what is required for moving forward. Tweets expressing support for older adults use the same pejorative terms (e.g., “boomer,” “oldies”), suggesting that advocacy for acceptable language use is a necessity.

Findings from our study have significant policy and practice implications. Specifically, our research found that Twitter is being used to disseminate ageist discourse such as victim-blaming, pejorative content, aggressive sentiments, and hate speech toward older adults during the COVID-19 pandemic. This ageist messaging negatively shapes and influences the public’s perceptions and actions toward older adults. Ageist discourse on social media may normalize aggressive sentiments that, in turn, increase older adults’ risk for violence, abuse, and harm. Consequently, it is imperative that the public, governments, and policy-makers denounce ageist rhetoric and aggressive messaging targeted toward older adults during the pandemic. Accountability measures and repercussions must be put in place to address issues of ageist hate speech, especially on social media. Educational COVID-19 campaigns and awareness programs on social media are needed to counter ageist sentiments and false information and work to unify society in the pandemic. Accordingly, urgent action is needed to denounce aggressive messaging, counter misinformation, and promote intergenerational unity during the COVID-19 pandemic.

### Limitations

Although our study sheds light on vaccine-related ageism during the pandemic, it is not without limitations. For example, a general limitation of Twitter research is the issue of representation since sociodemographic information is not collected (e.g., socioeconomic status, ethnicity, culture, etc.). Moreover, one must have access to a technological device such as a smartphone, tablet, or computer to tweet, which means that not all people (depending on, e.g., socioeconomic groups, geography— rural, remote, and urban, internet access, etc.) will be equally represented on Twitter (Colditz et al., 2018). Thus, it is difficult to make strong generalizations toward specific groups based on sociodemographic factors. However, existing data suggest that Twitter users worldwide are approximately 56% male and 44% female (Dixon, 2022b), and that the age groups of Twitter users include 13–17 years (6.6%), 18–24 years (17.1%), 25–34 years (38.5%), 35–49 years (20.7%), and 50 plus years (17.1%; Dixon, 2022c). Data also indicate that the top three leading countries of Twitter users (based on the number in millions) include the United States (77), Japan (59), and India (24; Dixon, 2022a). Further research is required to examine specific sociodemographic factors (such as socioeconomic status, culture, and ethnicity) in relation to vaccine-related ageism during the COVID-19 pandemic.

Another limitation of our study is that images (such as photos, emojis, and gifs) were not included in our analysis of the tweets. Although the use of images is a common practice in Twitter research, excluding images from our data set may remove some of the contextual information of the tweet such as visual illustrations to portray sarcasm, endearment, or humor.

In addition, because our Tweets were global and did not focus on a specific geographical region or country, it was sometimes challenging to identify cultural nuances related to the ageist labels used in the tweets. For example, the team had difficulty identifying the cultural nuances surrounding different labels such as “oldies” and whether this term depicted a form of stigmatizing endearment or sarcasm. Moreover, the rapidly evolving context and differences between jurisdictions in vaccine recommendations, policies, and product availability added additional layers of complexity to the interpretation of tweets. Consequently, the team analyzed each tweet independently to interpret the meaning and intention of each statement. Further research is required to examine ageist language and terminology within different cultures and regions, especially on social media platforms such as Twitter. Moreover, Twitter research conducted with a team of international researchers from diverse countries may provide a more comprehensive lens for interpreting cultural nuances and labels from different countries.

## Conclusion

The tweets analyzed in our research revealed extensive vaccine ageism during the COVID-19 pandemic. More specifically, our findings exposed issues of victim-blaming (e.g., it’s all their fault), hate speech (e.g., boomers need to die), pejorative content (e.g., okay boomer), and ageist political slander (e.g., senile old Joe) that is deepening the divide of intergenerational conflict. Moreover, our study findings suggest that ageism may have contributed to underlying fears and COVID-19 vaccine hesitancy among older adults.

Too often during the COVID-19 pandemic, ageist messaging has been considered an acceptable form of discourse and bias. Anonymity of social media enables users to assert hostile ageist sentiments and victim-blaming with little accountability or repercussions. Ageist tweets dehumanize older adults and often convey aggressive messaging that normalizes violence, harm, and abuse toward older adults. Accordingly, urgent action is needed to denounce aggressive messaging, counter misinformation, and promote intergenerational unity during the COVID-19 pandemic and beyond.

## Data Availability

(1) Supplementary materials (codebook) are available to other researchers for replication purposes; (2) supplementary data (Twitter excel sheet) are available by contacting the lead author; and (3) the study reported in the manuscript was not pre-registered.

## References

[CIT0001] Ambi, C. (2021). *How to scrape tweets without Twitter’s API using TWINT*. Medium. Retrieved January 17, 2022, from https://pub.towardsai.net/how-to-scrape-tweets-without-twitters-api-using-twint-797b196b951c

[CIT0002] Amnesty International Italia. (2022, August 8). *Italy: Violations of the human rights of older residents of care homes during COVID-19 pandemic*. Amnesty International. Retrieved January 20, 2022, from https://www.amnesty.org/en/latest/news/2020/12/italyviolations-of-the-h-uman-rights-of-older-residents-of-care-homes-during-covid-19-pandemic/

[CIT0003] Apriceno, M., Lytle, A., Monahan, C., Macdonald, J., & Levy, S. R. (2021). Prioritizing health care and employment resources during COVID-19: Roles of benevolent and hostile ageism. Gerontologist, 61(1), 98–102. doi:10.1093/geront/gnaa165 33119089 PMC7665451

[CIT0004] Bacsu, J. D., Fraser, S., Chasteen, A. L., Cammer, A., Grewal, K. S., Bechard, L. E., Bethell, J., Green, S., McGilton, K. S., Morgan, D., O’Rourke, H. M., Poole, L., Spiteri, R. J., & O’Connell, M. E. (2022a). Using Twitter to examine stigma against people with dementia during COVID-19: Infodemiology Study. JMIR Aging, 5(1), e35677. doi:10.2196/35677 35290197 PMC9015751

[CIT0005] Bacsu, J. R., O’Connell, M. E., & Wighton, M. B. (2022b). Improving the health equity and the human rights of Canadians with dementia through a social determinants approach: A call to action in the COVID-19 pandemic. Canadian Journal of Public Health—Revue Canadienne de Sante Publique, 113(2), 204–208. doi:10.17269/s41997-022-00618-8 35239172 PMC8892822

[CIT0006] Barrett, A. E., Michael, C., & Padavic, I. (2021). Calculated ageism: Generational sacrifice as a response to the COVID-19 pandemic. The Journals of Gerontology, Series B: Psychological Sciences and Social Sciences, 76(4), e201–e205. doi:10.1093/geronb/gbaa132 32841334 PMC7499750

[CIT0007] Binstock, R. H. (2010). From compassionate ageism to intergenerational conflict? Gerontologist, 50(5), 574–585. doi:10.1093/geront/gnq056 20837512

[CIT0008] Braun, V., & Clarke, V. (2006). Using thematic analysis in psychology. Qualitative Research in Psychology, 3(2), 77–101. doi:10.1191/1478088706qp063oa

[CIT0009] Braun, V., & Clarke, V. (2021). Thematic analysis a practical guide. Sage Publications Ltd.

[CIT0010] Cary, L. A., Chasteen, A. L., & Remedios, J. (2017). The Ambivalent Ageism Scale: Developing and validating a scale to measure benevolent and hostile ageism. Gerontologist, 57(2), e27–e36. doi:10.1093/geront/gnw118 27520730

[CIT0011] Centre for Ageing Better. (2021). *Dominant Narratives on Ageing.* https://ageing-better.org.uk/sites/default/files/2021-08/Dominant-narratives-ageing-full-report.pdf

[CIT0012] Chasteen, A. L., & Madey, S. F. (2003). Belief in a just world and the perceived injustice of dying young or old. Omega-Journal of Death and Dying, 47(4), 313–326. doi:10.2190/W7H7-TE9E-1FWN-B8XD

[CIT0013] Chasteen, A. L., Schwarz, N., & Park, D. C. (2002). The activation of aging stereotypes in younger and older adults. The Journals of Gerontology, Series B: Psychological Sciences and Social Sciences, 57(6), P540–P547. doi:10.1093/geronb/57.6.p540 12426437

[CIT0014] Colditz, J. B., Chu, K. H., Emery, S. L., Larkin, C. R., James, A. E., Welling, J., & Primack, B. A. (2018). Toward real-time infoveillance of Twitter health messages. American Journal of Public Health, 108(8), 1009–1014. doi:10.2105/AJPH.2018.304497 29927648 PMC6050832

[CIT0015] Dixon, S. (2022a). Countries with the most Twitter users 2022. Statista. Retrieved April 3, 2023, from https://www.statista.com/statistics/242606/number-of-active-twitter-users-in-selected-countries/?locale=en

[CIT0016] Dixon, S. (2022b). Distribution of Twitter users worldwide as of January 2022, by gender. Statista. Retrieved April 3, 2023, from https://www.statista.com/statistics/828092/distribution-of-users-on-twitter-worldwide-gender/?locale=en

[CIT0017] Dixon, S. (2022c). Twitter: Distribution of global audiences 2021, by age group. Retrieved April 5, 2023, from https://www.statista.com/statistics/283119/age-distribution-of-global-twitter-users/

[CIT0018] Duarte, A., & Albo, M. (2018, February 2). *Who you calling “Young Lady?”* American Association of Retired Persons. Retrieved April 11, 2023, from https://www.aarp.org/disrupt-aging/stories/ideas/info-2018/ageist-language-glossary.html

[CIT0019] Elliott, R. (2022). The “Boomer remover”: Intergenerational discounting, the coronavirus and climate change. The Sociological Review, 70(1), 74–91. doi:10.1177/00380261211049023

[CIT0020] Fiske, S. T., Cuddy, A. J., Glick, P., & Xu, J. (2002). A model of (often mixed) stereotype content: Competence and warmth respectively follow from perceived status and competition. Journal of Personality and Social Psychology, 82(6), 878–902. doi:10.1037/0022-3514.82.6.878 12051578

[CIT0021] Fraser, S., Lagacé, M., Bongué, B., Ndeye, N., Guyot, J., Bechard, L., Garcia, L., Taler, V., Adam, S., Beaulieu, M., Bergeron, C. D., Boudjemadi, V., Desmette, D., Donizzetti, A. R., Éthier, S., Garon, S., Gillis, M., Levasseur, M., Lortie-Lussier, M., & Tougas, F.; CCNA Social Inclusion and Stigma Working Group (2020). Ageism and COVID-19: What does our society’s response say about us? Age and Ageing, 49(5), 692–695. doi:10.1093/ageing/afaa097 32377666 PMC7239227

[CIT0022] Gendron, T. L., Welleford, E. A., Inker, J., & White, J. T. (2016). The language of ageism: Why we need to use words carefully. Gerontologist, 56(6), 997–1006. doi:10.1093/geront/gnv066 26185154

[CIT0023] Graham, M. E. (2022). “Remember this picture when you take more than you need”: Constructing morality through instrumental ageism in COVID-19 memes on social media. Journal of aging studies, 61, 101024. doi:10.1016/j.jaging.2022.101024 35654550 PMC8935246

[CIT0024] Horhota, M., Chasteen, A. L., & Crumley-Branyon, J. J. (2019). Is ageism acceptable when it comes from a familiar partner? The Journals of Gerontology, Series B: Psychological Sciences and Social Sciences, 74(4), 595–599. doi:10.1093/geronb/gby066 29846730

[CIT0025] Jimenez-Sotomayor, M. R., Gomez-Moreno, C., & Soto-Perez-de-Celis, E. (2020). Coronavirus, ageism, and Twitter: An evaluation of tweets about older adults and COVID-19. Journal of the American Geriatrics Society, 68(8), 1661–1665. doi:10.1111/jgs.16508 32338787 PMC7267430

[CIT0026] JMIR. (2022). *Do I need ethics approval for social media research?* https://support.jmir.org/hc/en-us/articles/115001620728-Do-I-need-ethics-approval-for-social-media-research-

[CIT0027] Levy, B. (2009). Stereotype embodiment: A psychosocial approach to aging. Current Directions in Psychological Science, 18(6), 332–336. doi:10.1111/j.1467-8721.2009.01662.x 20802838 PMC2927354

[CIT0028] Lloyd-Sherlock, P., Lasco, G., McKee, M., Perianayagam, A., & Sempé, L. (2021). Does vaccine ageism amount to gerontocide? Lancet, 398(10304), 952–953. doi:10.1016/S0140-6736(21)01689-5 34390657

[CIT0029] Meisner, B. A. (2021). Are you OK, Boomer? Intensification of ageism and intergenerational tensions on social media amid COVID-19. Leisure Sciences, 43(1–2), 56–61. doi:10.1080/01490400.2020.1773983

[CIT0030] Ng, R., Chow, T. Y. J., & Yang, W. (2021). Culture linked to increasing ageism during COVID-19: Evidence from a 10-billion-word corpus across 20 countries. The Journals of Gerontology, Series B: Psychological Sciences and Social Sciences, 76(9), 1808–1816. doi:10.1093/geronb/gbab057 33786581 PMC8083600

[CIT0031] Ng, R., & Indran, N. (2022). Hostility toward baby boomers on TikTok. Gerontologist, 62(8), 1196–1206. doi:10.1093/geront/gnac020 35106587 PMC9451021

[CIT0032] Ng, R., Indran, N., & Liu, L. (2022). Ageism on Twitter during the COVID-19 pandemic. The Journal of Social Issues, 78(4), 842–859. doi:10.1111/josi.12535 PMC9349453 35942488

[CIT0033] Ng, R., & Lim-Soh, J. W. (2021). Ageism linked to culture, not demographics: Evidence from an 8-billion-word corpus across 20 countries. The Journals of Gerontology, Series B: Psychological Sciences and Social Sciences, 76(9), 1791–1798. doi:10.1093/geronb/gbaa181 33099600 PMC8557828

[CIT0034] North, M. S., & Fiske, S. T. (2013). Act your (old) age: Prescriptive, ageist biases over succession, consumption, and identity. Personality & Social Psychology Bulletin, 39(6), 720–734. doi:10.1177/0146167213480043 23471317 PMC4486053

[CIT0035] O’Connor, C., & Joffe, H. (2020). Intercoder reliability in qualitative research: Debates and practical guidelines. International Journal of Qualitative Methods, 19(1), 1609406919899220. doi:10.1177/1609406919899220

[CIT0036] Previtali, F., Allen, L. D., & Varlamova, M. (2020). Not only virus spread: The diffusion of ageism during the outbreak of COVID-19. Journal of Aging & Social Policy, 32(4-5), 506–514. doi:10.1080/08959420.2020.1772002 32507060

[CIT0037] Pullen, C., Steele, J., & Patrick, J. H. (2021). Ageism predicts prioritizing COVID-19 vaccines for older adults and LTC residents. Innovation in Aging, 5(Suppl 1), 599–600. doi:10.1093/geroni/igab046.2301

[CIT0038] Raskind, I. G., Shelton, R. C., Comeau, D. L., Cooper, H. L. F., Griffith, D. M., & Kegler, M. C. (2019). A review of qualitative data analysis practices in health education and health behavior research. Health Education & Behavior, 46(1), 32–39. doi:10.1177/1090198118795019 30227078 PMC6386595

[CIT0039] Ravn, S., Barnwell, A., & Barbosa Neves, B. (2020). What is “Publicly Available Data”? Exploring blurred public–private boundaries and ethical practices through a case study on Instagram. Journal of Empirical Research on Human Research Ethics, 15(1-2), 40–45. doi:10.1177/1556264619850736 31132903

[CIT0040] Rowe, J. W. (2022). Covid-19 and aging: Challenges and opportunities. The Journals of Gerontology, Series A: Biological Sciences and Medical Sciences, 77(7), 1349–1351. doi:10.1093/gerona/glac089 35789373 PMC9384367

[CIT0041] Sipocz, D., Freeman, J. D., & Elton, J. (2021). “A toxic trend?”: Generational conflict and connectivity in Twitter discourse under the #BoomerRemover Hashtag. Gerontologist, 61(2), 166–175. doi:10.1093/geront/gnaa177 33159524 PMC7717318

[CIT0042] Skipper, A. D., & Rose, D. J. (2021). #BoomerRemover: COVID-19, ageism, and the intergenerational twitter response. Journal of Aging Studies, 57, 100929. doi:10.1016/j.jaging.2021.100929 34082999 PMC9758966

[CIT0043] Spaccatini, F., Giovannelli, I., & Pacilli, M. G. (2022). “You are stealing our present”: Younger people’s ageism towards older people predicts attitude towards age-based COVID-19 restriction measures. The Journal of Social Issues. doi:10.1111/josi.12537. Advance online publication. 10.1111/josi.12537 PMC9538229 36249551

[CIT0044] Swift, H. J., & Chasteen, A. L. (2021). Ageism in the time of COVID-19. Group Processes & Intergroup Relations: GPIR, 24(2), 246–252. doi:10.1177/1368430220983452 33746563 PMC7941503

[CIT0045] Takats, C., Kwan, A., Wormer, R., Goldman, D., Jones, H. E., & Romero, D. (2022). Ethical and methodological considerations of Twitter data for public health research: Systematic review. Journal of Medical Internet Research, 24(11), e40380. doi:10.2196/40380 36445739 PMC9748795

[CIT0046] Twitter: Number of users worldwide 2024. (2022, December 14). Statista. Retrieved December 14, 2022, from https://www.statista.com/statistics/303681/twitter-users-worldwide/

[CIT0047] Wilkerson, I. (2021). Caste: The origins of our discontents. Large print edition. Thorndike Press, a part of Gale, a Cengage Company.

[CIT0048] World Health Organization. (2021). *Global report on ageism*. World Health Organization. Retrieved December 10, 2022, from https://www.who.int/publications/i/item/9789240016866

[CIT0049] World Health Organization. (2023). COVID-19 mortality and progress towards vaccinating older adults—Worldwide, 2020–2022. Weekly Epidemiological Record, 98(05), 53–61. World Health Organization. Retrieved April 5, 2023 from https://apps.who.int/iris/handle/10665/365827. 10.15585/mmwr.mm7205a1 PMC9927068 36730046

[CIT0050] Xiang, X., Lu, X., Halavanau, A., Xue, J., Sun, Y., Lai, P. H. L., & Wu, Z. (2021). Modern senicide in the face of a pandemic: An examination of public discourse and sentiment about older adults and COVID-19 using machine learning. The Journals of Gerontology, Series B: Psychological Sciences and Social Sciences, 76(4), e190–e200. doi:10.1093/geronb/gbaa128 32785620 PMC7454882

